# Incidence and risk factors of inguinal hernia after robot-assisted radical prostatectomy

**DOI:** 10.1186/s12957-017-1126-3

**Published:** 2017-03-16

**Authors:** Yuta Yamada, Tetsuya Fujimura, Hiroshi Fukuhara, Toru Sugihara, Kotaro Takemura, Shigenori Kakutani, Motofumi Suzuki, Tohru Nakagawa, Haruki Kume, Yasuhiko Igawa, Yukio Homma

**Affiliations:** 1 Department of Urology, Graduate School of Medicine, The University of Tokyo, Hongo7-3-1, Bunkyo-ku, Tokyo, Japan; 2 Continence Medicine, Graduate School of Medicine, The University of Tokyo, Bunkyo-ku, Tokyo, Japan; 3 Department of Urology, Tokyo Teishin Hospital, Chiyoda-ku, Tokyo, Japan

**Keywords:** Inguinal hernia, Prostate cancer, Radical prostatectomy, Robot-assisted radical prostatectomy

## Abstract

**Background:**

Robot-assisted radical prostatectomy (RARP) has now become a gold standard approach in radical prostatectomy. The aim of this study was to investigate incidence and risk factors of inguinal hernia (IH) after RARP.

**Methods:**

This study included 307 consecutive men who underwent RARP for the treatment of prostate cancer from January 2011 to August 2015. The incidence of IH after RARP was investigated. Clinical and pathological factors were also investigated to assess relationship with development of postoperative IH.

**Results:**

Median follow-ups were 380 days, and median age of patients was 67 years. Incidence of IH was 11.3, 14.0, and 15.4% at 1, 2, and 3 years after RARP, respectively. Postoperative IH occurrence was significantly associated with low surgeon experience and postoperative incontinence at 3 or 6 months after surgery (*P* = 0.019, *P* = 0.002, and *P* = 0.016, respectively).

**Conclusions:**

Most of the IH occurred within the first 2 years with a rate of 14%. Incidence of IH after RARP was significantly associated with surgical experience and incontinence outcomes.

## Background

Inguinal hernia (IH) is recognized as one of the complications of conventional open retropubic radical prostatectomy (ORRP). Regan et al. first reported IH as a complication of ORRP with an incidence of 12% at 6 months after surgery [1]. Since then, a number of studies indicated high incidence of IH after ORRP [2–8]. In addition, various risk factors are suggested to be associated with IH after ORRP. These factors include lower body mass index (BMI), old age, and history of IH [3, 5, 9]. However, there are few studies reporting the incidence of postoperative IH after robot-assisted radical prostatectomy (RARP) [2, 10].

Our objective was to assess the incidence of postoperative IH in patients undergoing RARP and investigate relationships between clinico-pathological parameters and incidence of IH after RARP.

## Methods

### Patient characteristics

This study included 307 consecutive patients who underwent RARP for prostate cancer at The University of Tokyo Hospital between January 2011 and August 2015. Surgeries were carried out by multiple surgeons using the da Vinci-S® (Intuitive Surgical Incorporation, Sunnyvale, CA) with a transperitoneal, six-port technique which was described by Menon et al. [11]. Pneumoperitoneal pressure was maintained at 10 mmHg in standard procedure, while the pressure was elevated to 15 mmHg in procedures such as resection of the dorsal vein complex and nerve preservation. The clinico-pathological demographics of patients of the present study (RARP) were collected and were analyzed in relation to postsurgical IH.

Staging was performed according to the American Joint Committee on Cancer (AJCC) TNM staging system [12]. Continence was defined as pad-free or 0 g of urinary leakage. “Low surgeon experience” was defined as surgeon experiencing less than 40 cases of RARP surgeries. Standard protocol of follow-ups after discharge was visits at 2 weeks and 1, 3, 6, 12, and every 6 months thereafter. Patients were diagnosed with IH by computed tomography for definite diagnosis.

All the patients provided a written informed consent. This study was approved by the “Ethics Committee of the University of Tokyo Hospital” (#2283) and is in accordance with the Helsinki Declaration.

### Statistical analyses

Statistical software JMP® Pro version 12 (©2015 SAS Institute Inc., Cary, CA, USA) was used for statistical analysis. Pearson’s chi-square tests were used for evaluating association between categorical values and incidence of IH, except for values that showed frequency under 5 in which Fisher’s test were used. Significant factors in the univariate analysis and previously reported factors such as age, BMI, history of smoking, and history of IH repair were then evaluated for multivariate analysis using multiple logistic regression models. Kaplan-Meier curve showed the proportion of IH occurrence after RARP in the total population of this study. The date of surgery was defined as time zero, and the date that IH occurred was set as first endpoint. *P* value of <0.05 was defined as statistically significant in any statistical analysis performed in this study.

## Results

Clinical characteristics of 307 patients who underwent RARP at our hospital are presented in Table 1. Median age, preoperative prostate-specific antigen (PSA) level, resected prostate weight, and body mass index were 67 years (interquartile range (IQR); 63–71), 7.7 ng/ml (IQR; 5.6–11.2), 40 grams (IQR; 30–50), and 24 kg/m^2^ (IQR; 22–25), respectively. Median estimated blood loss, operative, and console time were 300 ml (IQR; 150–600), 237 min (IQR; 200–274), and 183 min (IQR; 152–216), respectively.

**Table 1:** Characteristics of patients undergoing robot-assisted radical prostatectomy (*N* = 307)

Variables		Median value (IQR) or *N* (%)
Age (years)		67 (63–71)
Preop-PSA (ng/ml)		7.7 (5.6–11.3)
BMI (kg/m^2^)		24 (22–25)
History of smoking	No	118 (38%)
	Yes	189 (61%)
Diabetes mellitus	Absent	263 (86%)
	Present	44 (14%)
Hypertension	Absent	141 (46%)
	Present	166 (54%)
Ischemic heart disease	Absent	276 (90%)
	Present	31 (10%)
D’Amico risk classification	Low	51 (17%)
	Intermediate-high	256 (83%)
Operative time (min)		237 (200–274)
Console time (min)		183 (152–216)
Blood loss (ml)		300 (150–600)
Nerve preservation	None	212 (69%)
	Unilateral	92 (30%)
	Bilateral	3 (1%)
Prostate weight (g)^a^		40 (30–50)
pT stage	pT2a–T2c	205 (67%)
	≥pT3a	102 (33%)
Extraprostatic extension	Absent	210 (68%)
	Present	97 (32%)
Resection margin	Negative	233 (76%)
	Positive	74 (24%)

*Preop* preoperative, *PSA* prostate-specific antigen, *BMI* body mass index

^a^Prostate weight refers to the weight of the resected prostate

A total of 30 men developed IH after RARP (Table 2). There were no significant differences in laterality. Direct hernia was observed in only one patient, and bilateral inguinal hernias were seen in 5 patients.

**Table 2:** Features of inguinal hernia (IH) after robot-assisted radical prostatectomy (RARP)

		Incidence of IH after RARP (%)
No. of postoperative hernias		30 (100%)
Laterality	Right	13 (43%)
	Left	12 (40%)
	Bilateral	5 (17%)
Type	Indirect	27 (90%)
	Direct	1 (3%)
	Unknown	2 (7%)
Size of hernia	<1 cm	4 (13%)
	1–3 cm	12 (40%)
	>3 cm	7 (23%)
	Unknown	7 (23%)

*IH* inguinal hernia, *RARP* robot-assisted radical prostatectomy

**Fig. 1:**
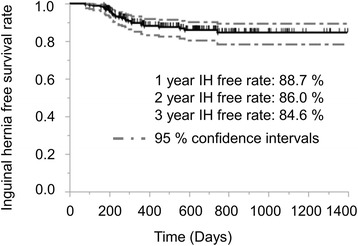
Kaplan-Meier curve showing proportion of inguinal hernia-free rates in patients with robot-assisted radical prostatectomy. Probability of IH-free survival after RARP is shown. IH-free rate was 88.7, 86.0, and 84.6% in the first, second, and third year

Kaplan-Meier curves showed proportion of IH-free rates in patients who underwent RARP (Fig. 1). Accumulative incidence of IH in patients with RARP was 11.3, 14.0, and 15.4% at 1, 2, and 3 years after surgery, respectively (Fig. 1).

In the univariate analysis regarding preoperative factors, age, BMI, history of smoking, and history of IH repair were not statistically significant factors associated with incidence of IH (Table 3). In addition, preoperative comorbidities such as hypertension and diabetes mellitus were also investigated but were not significant factors (Table 3). We then investigated postoperative factors and found that low surgeon experience and incontinence at 3 or 6 months were statistically significantly associated with the incidence of IH after RARP (*P* = 0.019, *P* = 0.002, and *P* = 0.016, respectively (Table 4)).

**Table 3:** Relationships between preoperative factors (clinical factors and comorbidities) and incidence of inguinal hernia (IH) developing after robot-assisted radical prostatectomy (RARP) (*N* = 307)

Preoperative clinical factors		Incidence of IH after RARP		
		No	Yes	*P* value
Age (years)	<67	132 (43%)	13 (5%)	0.653
	≥67	145 (47%)	17 (6%)	
Body mass index (kg/m2)	<24	92 (34%)	12 (4%)	0.820
	≥24	151 (55%)	18 (7%)	
Smoking history	Absent	104 (34%)	14 (5%)	0.337
	Present	172 (56%)	16 (5%)	
History of IH repair	Absent	268 (87%)	29 (9%)	0.980
	Present	9 (3%)	1 (1%)	
History of lower abdominal surgery	Absent	212 (69%)	25 (8%)	0.399
	Present	65 (21%)	5 (2%)	
Prostate-specific antigen (ng/ml)	<8	143 (46%)	18 (6%)	0.383
	≥8	134 (44%)	12 (4%)	
cT stage	cT1c	225 (73%)	24 (8%)	0.870
	≥cT2	52 (17%)	6 (2%)	
Preoperative comorbidities		No	Yes	P value
Cerebral vascular disease	Absent	266 (87%)	28 (9%)	0.369
	Present	11 (3%)	2 (1%)	
Hypertension	Absent	123 (40%)	18 (6%)	0.124
	Present	154 (50%)	12 (4%)	
Diabetes mellitus	Absent	237 (77%)	26 (9%)	1.000
	Present	40 (13%)	4 (1%)	
Hyperlipidemia	Absent	198 (64%)	19 (6%)	0.399
	Present	79 (26%)	11 (4%)	
Ischemic heart disease	Absent	248 (81%)	28 (9%)	0.752
	Present	29 (9%)	2 (2%)	

Pearson’s chi-square tests were used for statistical analyses except for “diabetes mellitus”, “history of IH repair”, “cerebral vascular disease”, and “ischemic heart disease” in which Fisher’s tests were used

**Table 4:** Relationships between surgical and pathological factors and incidence of inguinal hernia (IH) developing after robot-assisted radical prostatectomy (RARP) (*N* = 307)

Surgical and pathological factors		Incidence of IH after RARP		
		No	Yes	*P* value
Surgeon experience (cases)	Low (<40)	193 (63%)	27 (9%)	0.019
	High (≥40)	84 (27%)	3 (1%)	
Console time (min)	<183	137 (45%)	14 (5%)	0.771
	≥183	140 (46%)	16 (5%)	
Nerve sparing	None	193 (63%)	19 (6%)	0.475
	Performed^a^	84 (27%)	11 (4%)	
Lymph node dissection	None	219 (71%)	25 (8%)	0.582
	Performed	58 (19%)	5 (2%)	
Recovery of continence after surgery^b^	Absent	174 (56%)	27 (8%)	0.002
After surgery (at 3 months)^c^	Present	102 (33%)	3 (2%)	
Recovery of continence^b^	Absent	97 (39%)	19 (8%)	0.016
After surgery (at 6 months)^d^	Present	125 (50%)	9 (3%)	
Prostate weight^e^ (g)	<40	133 (43%)	17 (6%)	0.368
	≥40	144 (47%)	13 (4%)	
pT stage	≤pT2	181 (59%)	24 (8%)	0.106
	≥pT3	96 (31%)	6 (2%)	
Resection margin	Absent	209 (68%)	24 (8%)	0.580
	Present	68 (22%)	6 (2%)	

^a^Three were treated with bilateral nerve preservation, and the remaining 95 had unilateral nerve preservation

^b^“Recovery of continence” was defined as lack of necessity for using pads for urinary leakage

^c^ *N* = 306

^d^ *N* = 250

^e^Prostate weight refers to the weight of the resected prostate. Pearson’s chi-square tests were used for statistical analyses

Multivariate analysis showed that low surgical experience (<40 cases) and patients who were incontinent at 3 month were significant factors associated with the occurrence of IH (*P* = 0.004 and *P* < 0.001, Table 5).

**Table 5:** Multivariate analysis with respect to development of inguinal hernia after robot-assisted radical prostatectomy (*N* = 307)

Variables	Adjusted OR (95% CI)	*P* value
Age (≥67 vs <67 years)	1.02 (0.46–2.30)	0.960
Smoking history (No vs ≥yes)	1.55 (0.69–3.49)	0.287
BMI (<24 vs ≥24 kg/m^2^)	1.06 (0.46–2.38)	0.892
History of inguinal hernia repair (yes vs no)	1.03 (0.05–6.37)	0.976
Surgical experience (<40 vs ≥40 cases)	4.74 (1.57–20.56)	0.004
Continent at 3 months (no vs ≥yes)	6.41 (2.14–27.64)	<0.001

*OR* odds ratio, *CI* confidence interval, *BMI* body mass index

Continence after robot-assisted radical prostatectomy was defined as pad-free (complete continence)

Logistic regression model was used for multivariate analysis

*P* value of <0.05 was considered to be statistically significant

## Discussion

IH is regarded as one of the complications of ORRP. Incidence of IH after ORRP ranges from 12.2 to 23.9% during the first few years [2–8]. However, incidence of IH after RARP is not well known. To our knowledge, there are only two studies regarding incidence of IH after RARP [2, 10]. A European study revealed lower incidence of IH after RARP than after ORRP (12.2 vs. 5.8% at 48 months, respectively) [2]. A study by Lee et al. reported association between the presence of patent processus vaginalis (PPV) and development of IH in patients undergoing RARP [9]. The present study shows new findings that low surgeon experience, and continence outcomes are associated with the incidence of IH after RARP.

In general, IH occurs more frequently among men with low body mass index (BMI), old age, and history of smoking [13, 14]. Anatomically, the internal orifice is penetrated by the spermatic cord and is covered by the transversalis fascia, which act as a supportive structure in preventing development of IH. Patients with overweight have excessive fat and thickness of the abdominal wall that support the preventive effect against hernia formation. Aging may abate tension of the supporting connective tissue surrounding the internal inguinal ring [15]. It may also accelerate deterioration of muscular power of the abdominal muscles [13, 16]. Smoking is a known factor that is related to an increased risk of IH recurrence, since it causes change in the collagen composition by generating tissue hypoxia [14]. However, in the present study, low BMI, aging, and smoking were not significant factors related to the incidence of IH.

Previous reports have explored to identify other factors contributing to IH after radical prostatectomy (RP), whether the surgical types to be open surgery or not. Meta-analysis showed that increasing age, low BMI, previous inguinal hernia repair, presence of postoperative anastomotic stricture, and surgical types other than laparoscopic radical prostatectomy (LRP) or radical perineal prostatectomy were the significant factors predicting occurrence of IH [17]. In the present study, none of these factors were significantly associated with incidence of IH. However, one of the novel findings of our study was that development of IH was associated with persistent incontinence at 3 or 6 months after RARP. Factors attributing to IH and failure of gaining continence may be explained from the damage to the seamless pelvic structure including the transversalis fascia and pelvic floor muscles. Excessive damage to the pelvic floor may directly influence the condition of incontinence and, at the same time, weaken tension of the transversalis fascia leading to an increased risk of postoperative IH. In addition, not well-experienced surgeon may excessively spread the working space near the internal orifice when exposing the Retzius space, which may damage the transversalis fascia. This may be the reason to why low surgeon experience was significantly associated with the incidence of IH.

We have previously reported the incidence of IH in patients undergoing minimum incision endoscopic retropubic radical prostatectomy (MIES-RRP) and ORRP [18]. The incidence of IH after radical prostatectomy may be comparable between RARP and ORRP, since accumulative incidence of IH in patients with ORRP were 9.3% at 1 year and 13.1% at 2 years. However, it may be possible that IH may occur more frequently in patients with RARP than those with MIES-RRP, considering the fact that the accumulative incidence of IH in patients with MIES-RRP was 5.9% at 1 year and the same rate at 2 years. This is interesting since the procedures of MIES-RRP and RARP share commonality with the size of skin incision, and yet the incidence of IH seemed to be different. In MIES-RRP, surgeons have smaller limited working area due to the smaller skin incision. Whereas in RARP procedure, with its magnified vision and extended mobility, surgeons can easily achieve exposure with a wider operational area. Therefore, a smaller skin incision in MIES-RRP involves a limited exposed area attenuating the damage that may increase the risk for the development of IH. However, in RARP patients, small skin incision that is made for retrieving the prostate specimen does not affect the range and degree of exposure inside the abdomen, since this incision is made after prostatectomy. Unfortunately, a wider range of good exposure is potentially available in RARP procedure that may lead to an increased risk of developing postoperative IH. Further, based on this idea, it would be easy to understand the low incidence of IH development in patients who undergo laparoscopic radical prostatectomy [19, 20]. The limited mobility of laparoscopic instruments results in a relatively less dissection and damage to the transversalis fascia. To note, incidence of IH is surprisingly low with a range of 0–1.8% after radical perineal prostatectomy, in which the transversalis fascia is intact [21, 22].

In the future, studies demonstrating prophylactic methods may be required, since high incidence of IH is observed in RARP.

## Conclusions

The present study shows that the incidence of IH in RARP patients was similar with the previous reports. Interestingly, incidence of IH after RARP was significantly associated with incontinence outcomes.

## Abbreviations

BMI: Body mass index
IH: Inguinal hernia
MIES-RRP: Minimum incision endoscopic retropubic radical prostatectomy
ORRP: Conventional open retropubic radical prostatectomy
PSA: Prostate-specific antigen
PW: Prostate weight
RARP: Robot-assisted radical prostatectomy
RP: Radical prostatectomy
RRP: Retropubic radical prostatectomy
